# Current Status of *Trypanosoma grosi* and *Babesia microti* in Small Mammals in the Republic of Korea

**DOI:** 10.3390/ani14070989

**Published:** 2024-03-22

**Authors:** Hyun Jung Kim, BoGyeong Han, Hee-Il Lee, Jung-Won Ju, Hyun-Il Shin

**Affiliations:** Division of Vectors and Parasitic Diseases, Korea Disease Control and Prevention Agency, 187 Osongsaenmyeong 2-ro, Osong-eup, Heungdeok-gu, Cheongju 28159, Republic of Korea; kimhj0324@korea.kr (H.J.K.); borudd1@korea.kr (B.H.); isak@korea.kr (H.-I.L.); jupapa@korea.kr (J.-W.J.)

**Keywords:** wild animal, rodents, shrews, protozoa, molecular epidemiology

## Abstract

**Simple Summary:**

Parasitic protozoa are a common cause of vector-borne disease outbreaks and have been identified in different groups of wild animal species. Small mammals, such as rodents and shrews, play an important role in the transmission and maintenance of parasites. To understand the risk of parasitic protozoa, it is essential to have a good understanding of the status of transmitting vectors. This study aimed to investigate the diversity and current status of parasitic protozoa, including *Trypanosoma*, *Babesia*, and *Theileria*, from small mammals in the Republic of Korea. We found that the prevalence of *Trypanosoma grosi* was 23.9% (79/331) and *Babesia microti* was 10% (33/331), while *Theileria* was not detected in small mammals. These results can be used to raise awareness of parasite infection in the Republic of Korea.

**Abstract:**

Small mammals, such as rodents and shrews, are natural reservoir hosts of zoonotic diseases, including parasitic protozoa. To assess the risk of rodent-borne parasitic protozoa in the Republic of Korea (ROK), this study investigated the status of parasitic protozoa, namely *Trypanosoma*, *Babesia*, and *Theileria*, in small mammals. In total, 331 blood samples from small mammals were analyzed for parasites using PCR and sequenced. Samples were positive for *Trypanosoma grosi* (23.9%; *n* = 79) and *Babesia microti* (10%; *n* = 33) but not *Theileria*. Small mammals from Seogwipo-si showed the highest infection rate of *T. grosi* (48.4%), while the highest *B. microti* infection rate was observed in those from Gangneung-si (25.6%). Sequence data revealed *T. grosi* to be of the AKHA strain. Phylogenetic analysis of *B. microti* revealed the US and Kobe genotypes. *B. microti* US-type–infected small mammals were detected throughout the country, but the Kobe type was only detected in Seogwipo-si. To our knowledge, this is the first nationwide survey that confirmed *T. grosi* and *B. microti* infections at the species level in small mammals in the ROK and identified the Kobe type of *B. microti*. These results provide valuable information for further molecular epidemiological studies on these parasites.

## 1. Introduction

Small mammals, such as rodents and shrews, are well-known hosts and reservoirs of zoonotic diseases that pose a crucial threat to human health. Over 2000 species of rodents are distributed worldwide and live closely with humans [1,2]. Of these, approximately 200 species are reservoirs of more than 60 zoonotic diseases caused by viruses, bacteria, and parasites [3,4]. *Yersinia pestis*, *Salmonella*, and *Hantavirus* are among the most important pathogens affecting public health [5,6].

Zoonotic parasites are a common cause of vector-borne disease outbreaks and have been identified in different groups of wild animal species. Rodents play an important role in the transmission and maintenance of zoonotic parasites [6,7]. Most parasites in the blood and tissues of rodents can be transmitted to humans through contaminated food, water, rodent urine and feces, or via ectoparasites [8]. Therefore, it is necessary to investigate zoonotic parasite infections in small mammals to reduce exposure risk and predict future trends in pathogen prevalence and distribution according to seasonal and environmental changes.

Several zoonotic parasitic protozoa, such as *Trypanosoma* and *Babesia*, which cause trypanosomiasis and babesiosis, respectively, have gained importance as infectious agents. *Trypanosoma* is a parasitic hemoflagellate protozoan belonging to the Trypanosomatidae family that can infect animals and humans [9,10]. Certain species of this parasite may be transmitted by blood-feeding arthropods such as *Triatoma* sp. (kissing bug) and *Glossina* sp. (tsetse fly). *Trypanosoma* cause serious diseases in humans, such as Chagas disease (also known as American trypanosomiasis), caused by *Trypanosoma cruzi*, and African trypanosomiasis, caused by the *Trypanosoma brucei* complex [11].

Piroplasms are parasitic protozoa belonging to the genera *Babesia* and *Theileria*, which are the causative agents of babesiosis and theileriosis, respectively. Both intraerythrocytic protozoan parasites are transmitted by ticks [12,13] and are recognized for their important economic effects on the livestock industry and human health. Zoonotic babesiosis, mainly caused by *Babesia microti*, has recently posed a serious public health risk worldwide, in contrast to theileriosis, which has no zoonotic potential [14,15]. Approximately 100 *Babesia* species are known to infect animals and humans. The common clinical symptoms of babesiosis include fever, chills, fatigue, and headache [16,17]. The incidence of babesiosis has increased between 2011 and 2019 in the United States [18]. Cases of *Babesia* infections have been reported in various countries worldwide, including China, Germany, Canada, Australia, Japan, and the Republic of Korea (ROK) [19,20].

Therefore, given the importance of these three parasitic protozoa within the livestock industry and/or Public Health and as they can also be found in wild small mammals as reservoir hosts, we used molecular methods to investigate the diversity and current status of parasitic protozoa, including *Trypanosoma*, *Babesia*, and *Theileria*, in small mammals in the ROK.

## 2. Materials and Methods

### 2.1. Ethical Approval

All animal experiments were performed according to the guidelines for ethical conduct in the care and use of animals and approved by the Institutional Animal Care and Use Committee of the Korea Disease Control and Prevention Agency (approval number: KDCA-102-22). Permission to capture small mammals was obtained from each site in accordance with the Wildlife Protection and Management Act of the ROK.

### 2.2. Small Mammal Sampling

**Figure 1:**
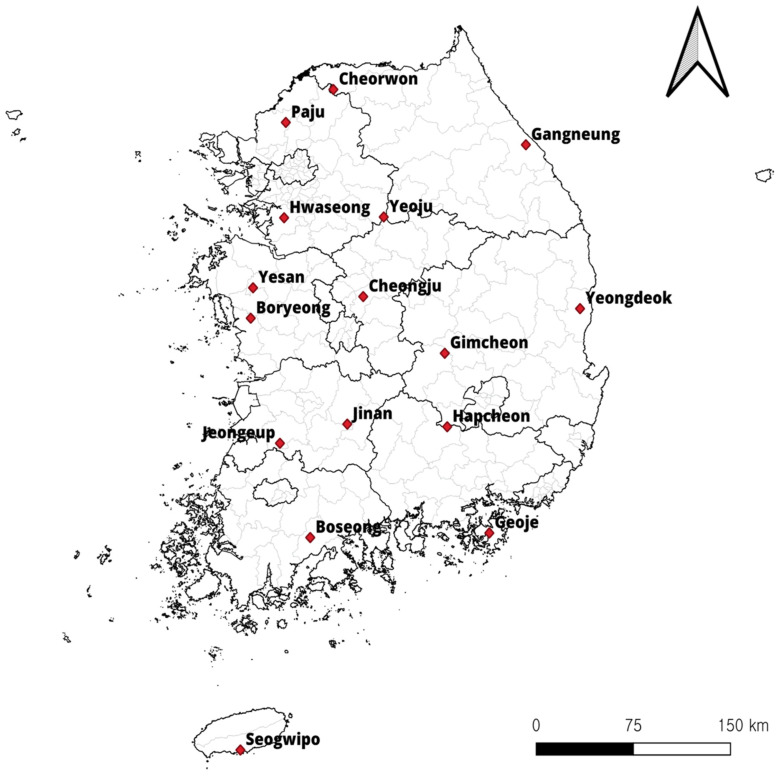
Trapping sites (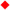) in the Republic of Korea (ROK). Map was created using the Free and Open Source QGIS (QGIS 3.28.10. Geographic Information System, http://www.qgis.org, accesed on 8 September 2023).

This study was conducted at 16 sites in the ROK in 2021 (Figure 1). Trapping was performed in the spring (March and April) and autumn (October and November). Small mammals were captured using Sherman folding live traps (BioQuip Products, Rancho Dominguez, CA, USA) baited with pieces of cheese crackers set in various habitats, including rice paddy fields, dry paddy fields, waterways, meadows, and reservoirs. Live small mammals were transferred to the laboratory and euthanized with carbon dioxide. After the identification of small mammals [2], blood was drawn from the heart using syringes, preserved in EDTA vacutainer tubes (BD, Franklin Lakes, NJ, USA), and stored at −20 °C until further use.

### 2.3. Molecular Detection of Parasites in Small Mammals

Genomic DNA was extracted from 331 small mammal blood samples (100 μL each) using the QIAamp DNA Blood Mini Kit, following the manufacturer’s protocol (Qiagen, Hilden, Germany). To detect parasite infection, we used the extracted DNA and amplified the *ITS1* gene of *Trypanosoma* spp. using TRYP1R (5′-GGAAGCCAAGTCATCCATCG-3′) and TRYP1S (5′-CGTCCCTGCCATTTGTACACAC-′3) primer sets targeting for ~623 bp fragments [21]. PCR was performed with 20 μL using an AccuPower PCR PreMix (Bioneer, Daejeon, Republic of Korea). Each PCR mixture was composed of 1 μL of each oligonucleotide primer (10 pmol/μL), 3 μL of genomic DNA as template, and 15 μL of distilled water. The conditions were initial denaturation at 95 °C for 5 min, followed by 35 cycles of 30 s at 95 °C, 1 min at 55 °C, and 1 min at 72 °C, with a final extension at 72 °C for 10 min in a C1000^TM^ Thermal Cycler (Bio-Rad, Hercules, CA, USA). Thus, we amplified the *18S rRNA* gene of *Babesia* and *Theileria* to obtain a 561 bp fragment using the commercial AccuPower Babesia & Theileria PCR Kit (Bioneer, Daejeon, Republic of Korea) according to the manufacturer’s instructions. Briefly, the lyophilized premix and primer comprised 3 μL genomic DNA as template, and 17 μL distilled water. The PCR reaction mixture was subjected to thermal cycling at 95 °C for 5 min, followed by 40 cycles of 20 s at 95 °C, and 50 s at 59 °C, with a final extension at 72 °C for 5 min. The genomic DNA of *B. microti* and *T. cruzi*, obtained from American Type Culture Collection (ATCC, Manassas, VA, USA), and *Theileria*, obtained from Bioneer (Daejeon, Republic of Korea), were used as positive controls. A suitable positive control and negative control were included in each amplification reaction. Amplification products were analyzed using a QIAxcel capillary electrophoresis system (Qiagen). The DNA extraction, PCR amplification, and automated electrophoresis were performed in separate rooms to prevent contamination.

### 2.4. Sequencing and Phylogenetic Analysis

Positive PCR products were purified using QIAquick PCR purification kits (Qiagen) and sequenced using Sanger sequencing and an ABI PRISM 3730xl analyzer (Life Technologies, Carlsbad, CA, USA). The nucleotide sequences were compared with reference sequences obtained from GenBank using nucleotide BLAST (National Center for Biotechnology Information, NCBI). All sequences used in phylogenetic analyses were downloaded from GenBank, available through the NCBI. A phylogenetic tree was constructed using the neighbor-joining method and p-distance model in MEGA X (Pennsylvania State University, State College, PA, USA). The sequences obtained in this study were submitted to GenBank (accession numbers: OP804252, OP804253, OP804254, OP297200, and OP297201).

### 2.5. Statistical Analysis

Statistical analyses were performed using IBM SPSS Statistics software version 26 (IBM, Armonk, NY, USA). Pearson’s chi-square test or Fisher’s exact test was used to examine the association between parasitic infections in small mammals and related factors, including small mammal species, seasonal habitat type, and trapping site. *p* < 0.05 was considered significant.

## 3. Results

### 3.1. Prevalence of Parasitic Protozoa in Small Mammals in the ROK

Over the two trapping seasons in 2021, 331 small mammals belonging to eight genera and ten species were collected from 16 sites in the ROK. The results showed that 14 sites harbored parasitic protozoa, *T. grosi* or *B. microti*, infecting small mammals, with the exceptions being Boryeong-si and Cheongju-si (Table 1); *Theileria* was not detected in any of the samples. The small mammals collected from Seogwipo-si had the highest *T. grosi* infection rate at 48.4% (15/31). The results also showed that 10 sites harbored *B. microti,* with the highest *B. microti* infection rate being 32.6% (14/43) in Gangneung-si. The infection rates in Seogwipo-si and Gangneung-si were significantly higher than those in other sites (*p* < 0.05). Also, our results showed that one genotype of *B. microti* was confirmed in each site. Among positive samples of *B. microti*, the US type was found to be the dominant genotype in the ROK; only the *B. microti* Kobe type was detected in Seogwipo-si, which is the first confirmation from small mammals in the ROK (Table 1).

### 3.2. Prevalence and Phylogenetic Analysis of T. grosi and B. microti in Small Mammals

Three species, *Apodemus agrarius* (striped field mouse), *Crocidura* sp., and *Apodemus peninsulae* (Korean field mouse), were confirmed to be infected with parasitic protozoa. *A. agrarius* was highly dominant, accounting for 87.6% (290/331) of trapped small mammals, and co-infected with *T. grosi* and *B. microti* in six individuals (Table 2).

**Figure 2:**
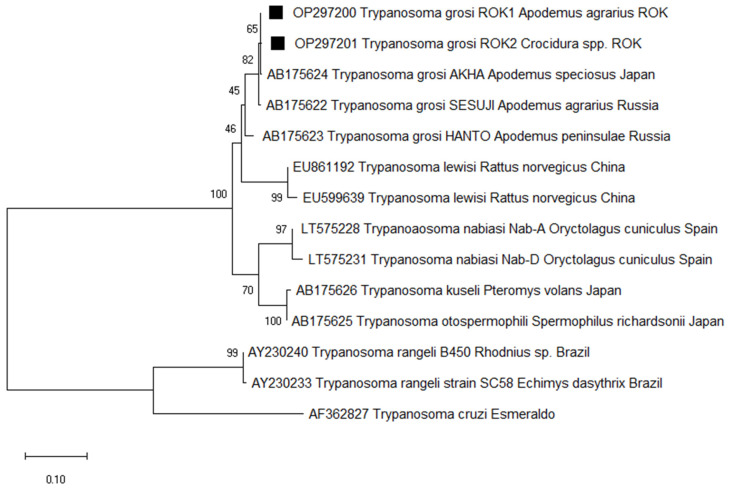
Phylogenetic tree based on neighbor-joining analysis of *internal transcriptional spacer 1* (*ITS1*) from GenBank and *Trypanosoma grosi*-positive small mammal specimens captured in the ROK in 2021. *Trypanosoma grosi* sequences obtained in this study are denoted by a solid square (■, GenBank accession numbers OP297200 and OP297201). The numbers on the branches indicate bootstrap percentages based on 1000 replication.

To detect *Trypanosoma* in small mammals, we target the *ITS1* gene of the protozoa. Of the 331 samples, 79 (23.9%) were positive and sequencing revealed 98.8% identity with *T. grosi* (AB175624), identified in two species of small mammals: *A. agrarius* (26.5%) and *Crocidura* sp. (8.7%). Of the 79 sequences identified as *T. grosi*, two representative sequences were selected without duplicates of sequences and host source. Therefore, the two *T. grosi ITS1* sequences were deposited in GenBank under the accession numbers OP297200 and OP297201. Phylogenetic analysis revealed that OP297200 (from *A. agrarius*) and OP297201 (from *Crocidura* sp.) are closely related with the previously reported *T. grosi* AKHA strain sequence (AB175624), which was isolated from *A. speciosus* in Japan (Figure 2).

**Figure 3:**
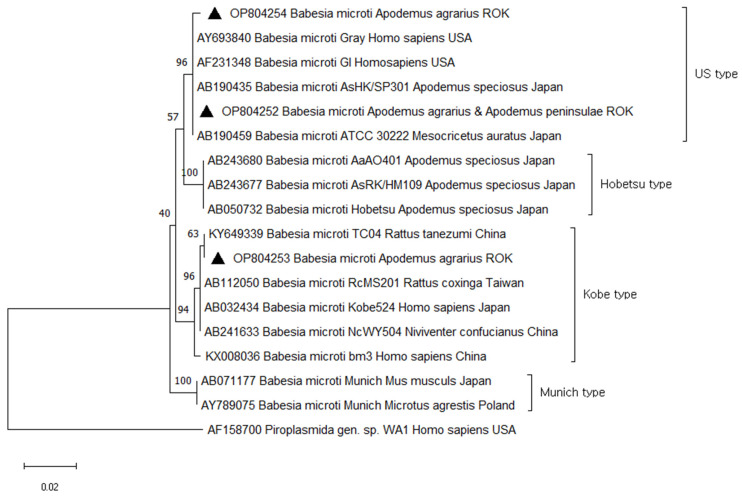
Phylogenetic tree based on neighbor-joining analysis of *Babesia 18S rRNA* from GenBank and *Babesia microti*-positive small mammal specimens captured in the ROK in 2021. *Babesia microti* sequences obtained in this study are denoted by a solid triangle (▲, GenBank accession numbers OP804252–OP804254). The numbers on the branches indicate bootstrap percentages based on 1000 replication.

A total of 33 (10.0%) samples were positive and sequencing revealed 100.0% similarity to *B. microti*, found in two species of small mammals: *A. agrarius* (11%) and *A. peninsulae* (50%). Of the 33 sequences identified as *B. microti*, three representative sequences were selected without duplicates of sequences and host source. The three *B. microti 18S rRNA* sequences were deposited in GenBank under accession numbers OP804252, OP804253, and OP804254. Phylogenetic analysis revealed that OP804252 (from *A. agrarius* and *A. peninsulae*) OP804254 (from *A. agrarius*) were closely related to *B. microti* US type (AY693840, AF231348, AB190435, and AB190459). These clusters contained *B. microti* US type that have been isolated from small mammals or humans in different regions, including USA (AY693840 and AF231348 from human) and Japan (AB190435 from *A. speciosus* and AB190459 from *M. auratus*). OP804253 (from *A. agrarius*) belonged to the *B. microti* Kobe type (KY649339 from *R. tanezumi*, AB112050 from *R. coxinga*, AB032434 from Human, AB241633 from *N. confucianus*, and KX008036 from human) (Figure 3).

### 3.3. Relationships between Season, Ecological Habitat, and T. grosi and B. microti Infections

In ecological habitats, the prevalence of *T. grosi* in small mammals from reservoirs was significantly lower than that in small mammals from other habitats (rice paddy fields, dry paddy fields, watery ways, and meadows; *p* = 0.006). The seasonal *T. grosi* positivity rate was higher in spring (26.4%; 46/174) than in autumn (21.0%; 33/157); however, the difference was not significant. The infection rate of *T. grosi* in *A. agrarius* was significantly higher than that in other species (*p* = 0.0014). The infection rates of *B. microti* per ecological habitat, season, and small mammal species are shown in Table 3. The seasonal *B. microti* positivity rate was 9.8% (17/174) for small mammals in spring and 10.2% (16/157) in autumn. There were no significant differences between seasons, small mammal species, and ecological habitats with respect to *B. microti* infection. However, *B. microti* DNA from small mammals was consistently detected throughout the study period in all habitats (Table 3).

## 4. Discussion

This study was a nationwide investigation of parasitic protozoa, including *Trypanosoma*, *Babesia*, and *Theileria*, in small mammals from 16 sites across the ROK and demonstrates a wide prevalence of parasitic protozoa. Over two trapping seasons in 2021, 331 small mammals belonging to eight genera and ten species were collected from 16 sites. Blood samples from small mammals were analyzed for parasites using PCR and positive samples were sequenced. Samples were positive for *Trypanosoma grosi* (23.9%) and *Babesia microti* (10.0%) but not *Theileria*. These results suggest that the diversity of parasitic protozoa in the ROK is high and even new species can be involved in the infections.

*Trypanosoma* is a flagellate blood parasite found in every vertebrate class [22,23]. Over 500 species of *Trypanosoma* have been recorded worldwide [24,25,26]. Trypanosomes can be categorized into two groups based on their transmission route from the vector to the host [27]. The first group, known as salivarian trypanosomes, includes *T. brucei*, the causative agent of human African trypanosomiasis; this species develops in the midgut of the vector, migrates to the salivary glands or proboscis, and is transmitted to the host through the vector’s saliva during the biting process. The second group, stercorarian trypanosomes, includes *T. cruzi*, the causative agent of Chagas disease. Stercorarian trypanosomes develop in the hindgut of the vector and are transmitted to the host either through the ingestion of vectors, such as fleas, or the contamination of bite wounds with vector feces [26,27]. A total of 44 *Trypanosoma* species have been identified in 144 rodent species, the majority of which belong to the stercorarian group [11,27]. Most of stercorarian trypanosomes are considered nonpathogenic, except for *T. cruzi*. To date, there has been no investigation of wild small mammals infected with *Trypanosoma* in the ROK, resulting in a critical knowledge gap.

*Trypanosoma grosi* belongs to the nonpathogenic stercorarian group [23,26] and has been detected in species of *Apodemus* from several countries, including Russia (*A. sylvaticus*), France (*A. sylvaticus*), Japan (*A. speciosus*), and China (*A. agrarius*) [26,28,29,30]. To the best of our knowledge, *T. grosi* has not yet been reported in small mammals in the ROK. Our results revealed that there were two species of small mammals (*A. agrarius* and *Crocidura* sp.) infected with *T. grosi* in the ROK, with a high infection rate of 23.9% compared to other protozoa such as *Babesia* and *Theileria*. This is also the first confirmed report of co-infection of *B. microti* and *T. grosi* in small mammals at the molecular level. To date, there has been no evidence of human infection with *T. grosi,* however, several cases have been reported of humans infected with *T. lewisi* or *T. lewisi*-like (*T. grosi*-involved group) [31,32,33]. Although *T. grosi* is harmless in humans, further investigation to maximize the understanding of these diseases should be considered. This study may be helpful in future molecular epidemiological studies of *T. grosi* in the ROK.

*Babesia* and *Theileria* are parasitic protozoa, mainly transmitted by ticks, that can infect a variety of domestic and wild animals. The prevalence of *Babesia* in small mammals was as high as 20.8% (32/154) in the ROK in 2001 [34]. However, there was a low infection rate of 0.52% (3/578) in 2008, reported for two districts (Gangwon-do and Gyeonggi-do) [35]. Both studies only detected the *B. microti* US type in small mammals. Compared to previous studies, our study showed that the infection rate of *Babesia* in small mammals in the ROK decreased from 20.8% in 2001 to 10.0% in 2021. Additionally, previous studies only confirmed the *B. microti* US type, but we used molecular methods to target *Babesia 18S rRNA* from small mammals in the ROK to identify two genetic groups of *B. microti*: the US and Kobe types. *B. microti* US-type–infected small mammals were distributed across various sites of the ROK, whereas *B. microti* Kobe-type–infected small mammals were detected only in Seogwipo-si. However, molecular evidence of *Babesia* genera in ticks has already been reported in the ROK, such as *B. duncani*, *B. venatorum*, *B. divergens*, and *B. microti* US type [36,37,38,39]. According to a recent report, the US type of *B. microti* was detected in ticks from small mammals in the ROK in 2017, while the Kobe type was only detected in ticks from the southern region of the ROK (Goheung-gun and Jeju island) [40]. This result is similar to that of our study, which showed that the Kobe type of *B. microti* was only detected in small mammals collected from Seogwipo-si, Jeju island. In the ROK, the first report of human babesiosis was reported in 1988. Around 16 cases of human babesiosis have been recorded since, most of which were imported from other countries [41,42]. Two cases were known to be locally infected with *Babesia* sp. KO-1 and *B. motasi*-like [37,43]. To date, these studies have revealed that there have been no cases of human infection by vectors transmitting *B. microti* in the ROK. Therefore, this study reinforces the importance of further studies addressing changes in *Babesia microti* genotypes in the study area.

Some species of *Theileria* such as *T. annulata* and *T. parva* are pathogenic to livestock and cause high rates of mortality in sheep and cattle [44]. Other *Theileria* spp. are thought to be less virulent, probably because of evolutionary interactions between *Theileria* and the host [45]. Recently, several studies have investigated the presence of *Theileria* spp. in various wild animals, including rodents. For example, *Theileria peramelis* was identified in *Rattus* (black rat) and *Perameles nasuta* (long-nosed bandicoots) in Australia [46]. *Theileria* sp. were successfully amplified from the rodents *Le. Striatus* and *Praomys* sp. in Gabon [47]. Those findings have not been confirmed for the zoonotic potential of *Theileria*. Therefore, further research is needed to investigate the potential consequences of infection in wildlife, especially the effect of pathogens spreading to native wildlife.

Our study has a few limitations: (1) we did not double-check positivity of *T. grosi* and/or *B. microti* in small mammals through microscopic examination. The difficulty with identifying species-level parasitic protozoa by microscopy has been described in several studies. Therefore, we used molecular methods to investigate the diversity and current status of parasitic protozoa at the species level in the small mammals. (2) only blood samples from small mammals were used to investigate the prevalence of parasitic protozoa. Further studies including the diversity and rate of parasitic infection from ectoparasites (such as fleas and ticks) in small mammals should be conducted to better understand these parasitic protozoa circulating in small mammals.

In this study, 33 of 331 samples (10.0%) were positive for *B. microti* in two small mammal species, *A. agrarius* and *A. peninsula*. These two small mammal species were confirmed to play an important role as reservoirs of *B. microti*, especially because *A. agrarius* is abundant and widely distributed across the country [48]. Although no statistical differences were observed between *B. microti* infection and season or habitat, *B. microti* DNA in small mammals was consistently detected over the study period and sites. Therefore, our findings suggest that small mammals infected with *T. grosi* and *B. microti* are widely and non-seasonally distributed throughout the ROK; however, there is no detection of *Theileria*. Additionally, this is the first study to detect *B. microti* Kobe type in small mammals in the ROK. These results provide useful information for further molecular epidemiological studies on parasites, especially those on *T. grosi* and *B. microti* transmission to humans or animals via vectors.

## 5. Conclusions

This study aimed to investigate the diversity and status of parasitic protozoa, including *Trypanosoma grosi*, *Babesia microti*, and *Theileria*, in small mammals in the ROK. Here, we present the rate of parasitic protozoa infection in small mammals in the study area. *T. grosi* was detected in small mammals for the first time. Furthermore, *B. microti* US-type–infected small mammals were detected throughout the country, but the Kobe type was only detected in Seogwipo-si. These results provide valuable information for further molecular epidemiological studies on these parasites.

## Figures and Tables

**Table 1:** Molecular prevalence of *Trypanosoma grosi* and *Babesia microti* and genetic diversity of *B. microti* in small mammals collected from different sites of the Republic of Korea (ROK) in 2021.

Site	No. Tested	No. *T. grosi-* Positive (%)	No. *B. microti-* Positive (%)	*B. microti* Genotype
Gangneung-si	43	11 (25.6)	14 (32.6) ^§§^	US type
Hwaseong-si	33	12 (36.4)	1 (3.0)	US type
Seogwipo-si	31	15 (48.4) ^§^	2 (6.5)	Kobe type
Jinan-gun	24	5 (20.8)	2 (8.3)	US type
Cheorwon-gun	23	6 (64.7)	1 (4.3)	US type
Geoje-si	23	6 (26.1)	0 (0.0)	
Yeoju-si	22	3 (13.6)	0 (0.0)	
Yeongdeok-gun	20	4 (20.0)	2 (10.0)	US type
Paju-si	19	6 (31.6)	6 (31.6)	US type
Jeongeup-si	18	1 (5.6)	2 (11.1)	US type
Gimcheon-si	18	1 (5.6)	0 (0.0)	
Boseong-gun	17	1 (5.9)	1 (5.9)	US type
Hapcheon-gun	16	1 (6.3)	2 (12.5)	US type
Yesan-si	14	2 (14.3)	0 (0.0)	
Boryeong-si	8	0 (0.0)	0 (0.0)	
Cheongju-si	8	0 (0.0)	0 (0.0)	
Total	331	79 (23.9)	33 (10.0)	

^§^ Significantly higher *T. grosi* infection rate in Seogwipo-si vs. other sites; ^§§^ Significantly higher *B. microti* infection rate in Gangneung-si vs. other sites.

**Table 2:** Prevalence of *Trypanosoma grosi* and *Babesia microti* in blood samples from small mammals collected from the ROK in 2021.

Small Mammal Species	No. Tested	No. of *T. grosi-* Positive (%)	No. of *B. microti-* Positive (%)	No. of Coinfection (%)
*Apodemus agrarius*	290	77 (26.5)	32 (11.0)	6 (2.0)
*Crocidura* sp.	23	2 (8.7)	0	0
*Apodemus peninsulae*	2	0	1 (50.0)	0
*Craseomys regulus*	4	0	0	0
*Craseomys rufocanus*	1	0	0	0
*Cricetulus triton*	1	0	0	0
*Micromys minutus*	6	0	0	0
*Microtus fortis*	2	0	0	0
*Myodes regulus*	1	0	0	0
*Rattus norvegicus*	1	0	0	0
Total	331	79 (23.9)	33 (10.0)	6 (1.8)

**Table 3:** Relationship between season, ecological habitat, and *Trypanosoma grosi* and *Babesia microti* infections in small mammals collected from the ROK in 2021.

Categories		No. Tested	No. of *T. grosi-* Positive (%)	*p*-Value	No. of *B. microti-* Positive (%)	*p*-Value
Season	Spring	174	46 (26.4)	0.248	17 (9.8)	0.898
	Autumn	157	33 (21.0)		16 (10.2)	
Ecological habitat	Reservoir	70	8 (11.4)	0.006	9 (12.8)	0.363
	Other	261	71(27.2)		24(9.2)	
Small mammal species	*A. agrarius*	290	77 (26.5)	0.0014	32 (11.0)	0.098
	Other	41	2 (4.8)		1 (2.4)	

## Data Availability

The sequence data generated in the current study are available in the GenBank repository under the accession numbers OP804252, OP804253, OP804254, OP297200, and OP297201. The datasets used and/or analyzed during the present study are available upon reasonable request.
